# Therapeutic Implications of Mesenchymal Stromal Cells and Their Extracellular Vesicles in Autoimmune Diseases: From Biology to Clinical Applications

**DOI:** 10.3390/ijms221810132

**Published:** 2021-09-20

**Authors:** Angelos Matheakakis, Aristea Batsali, Helen A. Papadaki, Charalampos G. Pontikoglou

**Affiliations:** 1 Department of Hematology, School of Medicine, University of Crete, 71500 Heraklion, Greece; a.matheakakis@gmail.com (A.M.); e.papadaki@uoc.gr (H.A.P.); 2 Haemopoiesis Research Laboratory, School of Medicine, University of Crete, 71500 Heraklion, Greece; tea_ios@yahoo.gr

**Keywords:** mesenchymal stromal cells, exosomes, extracellular vesicles, autoimmune disorders, immunomodulation

## Abstract

Mesenchymal stromal cells (MSCs) are perivascular multipotent stem cells originally identified in the bone marrow (BM) stroma and subsequently in virtually all vascularized tissues. Because of their ability to differentiate into various mesodermal lineages, their trophic properties, homing capacity, and immunomodulatory functions, MSCs have emerged as attractive candidates in tissue repair and treatment of autoimmune disorders. Accumulating evidence suggests that the beneficial effects of MSCs may be primarily mediated via a number of paracrine-acting soluble factors and extracellular vesicles (EVs). EVs are membrane-coated vesicles that are increasingly being acknowledged as playing a key role in intercellular communication via their capacity to carry and deliver their cargo, consisting of proteins, nucleic acids, and lipids to recipient cells. MSC-EVs recapitulate the functions of the cells they originate, including immunoregulatory effects but do not seem to be associated with the limitations and concerns of cell-based therapies, thereby emerging as an appealing alternative therapeutic option in immune-mediated disorders. In the present review, the biology of MSCs will be outlined and an overview of their immunomodulatory functions will be provided. In addition, current knowledge on the features of MSC-EVs and their immunoregulatory potential will be summarized. Finally, therapeutic applications of MSCs and MSC-EVs in autoimmune disorders will be discussed.

## 1. Introduction

Knowledge regarding the etiology, pathophysiology, and clinical manifestations of autoimmune disorders has witnessed considerable progress during the last years and this has paved the way for the development of sophisticated treatments targeting molecular pathways and immune deregulations implicated in disease pathogenesis. One such novel therapeutic modality involves the use of mesenchymal stromal sells (MSCs) [1].

MSCs are multipotent cells deriving from the mesoderm that can be isolated from various tissues with minimally invasive procedures [2,3]. Because of their potential to differentiate into several tissues, their extensive in vitro expansion, and their broad immunoregulatory properties, involving cells associated with both innate and adaptive immunity, MSCs have drawn much attention in the field of tissue repair [2,3,4]. Ιn support of this, several preclinical and clinical studies evaluating the immunomodulatory role of MSCs have indeed demonstrated promising results in terms of attenuating inflammatory and autoimmune disorders [1].

A rapidly growing number of studies suggests that the beneficial therapeutic and immunoregulatory functions of MSCs may be primarily mediated via a number of paracrine-acting soluble molecules and extracellular vesicles (EVs) and to a lesser extent by cell–cell contact [5]. EVs are membrane-coated vesicles that have emerged as important players in intercellular communication via their capacity to carry and deliver their cargo, consisting of proteins, nucleic acids, and lipids to target cells [5]. Interestingly, MSC-EVs have been shown to recapitulate the functions of the cells they originate, including immunoregulatory effects [6,7,8], whereas, in contrast to bona fide MSCs, they are not associated with the limitations and concerns of cell-based therapies, thereby emerging as an appealing alternative therapeutic option in immune-mediated disorders

In the present review, we attempt to outline the biology of MSCs and provide an overview of their immunomodulatory functions. In addition, we summarize the current knowledge on the features of MSC-derived EVs and their immunoregulatory potential. Finally, therapeutic applications of MSCs and MSC-EVs in autoimmune disorders will be discussed.

## 2. MSC Characteristics and Immunomodulatory Properties

Mesenchymal stromal cells (MSCs) are non-hematopoietic cells that were originally identified in the bone marrow (BM) by Alexander Friedenstein et al. in the late 1960s to early 1970s [2,9,10,11]. Via a series of pioneering experiments, the authors demonstrated that after seeding BM cells at low density, colonies of plastic-adherent fibroblast-like cells were formed. Each colony was derived from a single cell that was called colony-forming unit-fibroblast (CFU-F). Subcutaneous transplantation of the clonal progeny of a single CFU-F could give rise to fibrous tissue, bone, and bone containing marrow in the host [2,9,10,11]. Subsequent studies from other groups substantiated these findings and provided evidence that the cells isolated by Friedenstein and his colleagues were multipotent as they could differentiate into osteoblasts, adipocytes, and chondrocytes (reviewed in [12]).

In 2006, the International Society for Cellular Therapy (ISCT) established three minimal criteria for the definition of human MSCs [13]: (a) plastic adherence, (b) expression of the surface antigens CD73, CD90, CD105 while lacking the expression of the hematopoietic and endothelial molecules CD34, CD45, CD14, CD19, CD79a, CD11b, and HLA-DR (c) in vitro differentiation into three mesodermal lineages (osteoblasts, adipocytes, and chondrocytes). Certain authors have suggested that the differentiation capacity of MSCs may be broader, including even cells of non-mesodermal origin [14,15,16,17,18,19]. However, it is generally agreed that such differentiation potential has not been adequately substantiated [20].

Following the initial isolation of MSCs from the BM, a number of studies suggested that cells fulfilling the aforementioned criteria and sharing similar, but not identical, properties can be harvested from a wide variety of human tissues such as adipose tissue, dental pulp, peripheral blood, menstrual blood, endometrium, as well as fetal tissues including amniotic fluid, placenta, umbilical cord, Wharton jelly, and umbilical cord blood [21,22,23,24,25,26,27,28,29,30,31]. The detection of MSCs in multiple organs and tissues may reflect their perivascular in vivo localization. In fact, accumulating evidence suggests that native MSCs may derive from cells associated with blood vessels, namely pericytes and adventitial cells [32]. Thus, any vascularized tissue or organ would be expected to contain MSCs.

**Figure 1:**
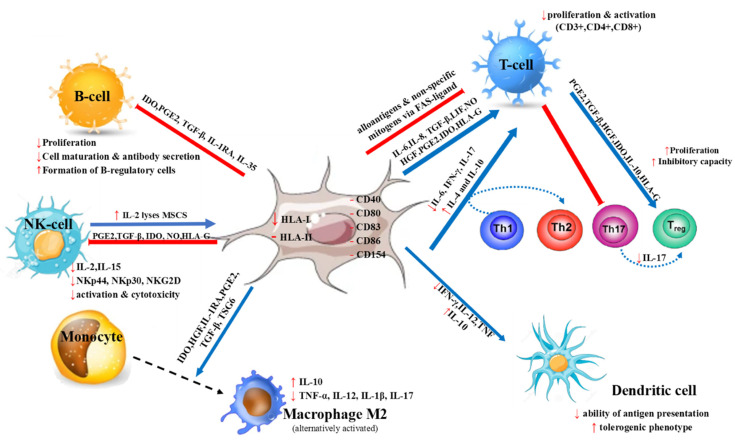
Interaction of MSCs with cells involved in innate and adaptive immune responses. The immunomodulatory effects of MSCs are mediated via cell–cell contact and secretion of soluble factors. MSC: Mesenchymal stromal cells.

A crucial property of MSCs is their low immunogenicity [2,3,6,33], as suggested by the low expression of HLA class I and the lack of expression of HLA class II and co-stimulatory molecules, including CD40, CD80, CD83, CD86, and CD154. This notion is further supported by the observation that MSCs do not induce a proliferative response from allogeneic lymphocytes [2,3,6,33] (Figure 1). Interestingly, inflammatory factors, i.e., interleukin-1β (IL-1β), interferon-gamma (IFN-γ), or tumor necrosis factor-α (TNF-α) result in upregulation of HLA class I and induction of expression of HLA class II antigens by MSCs [34], thereby potentiating their interactions with T cells [3,35,36,37]. Yet, as these factors have no effect on co-stimulatory molecules, T cells are not properly activated and eventually become anergic, even in the context of inflammation [3,38]. Nevertheless, some studies have shown that allogeneic MSCs can actually activate T cells [39] and it has also been demonstrated that infused MSCs into allogeneic major histocompatibility complex (MHC)-mismatched mice were rapidly rejected [2,40,41]. These findings suggest that MSCs may not be immune-privileged as originally thought; rather, in the allogeneic setting, MSCs should be regarded as hypoimmunogenic as compared to other cell types [2,3,39].

MSCs have been found to inhibit T lymphocyte proliferation and activation (Figure 1) in response to alloantigens, nonspecific mitogens, and specific antigens in vitro [2,36,41,42]. Immunosuppression includes all CD3^+^, CD4^+^, and CD8^+^ T subpopulations, can be exerted by both allogeneic and autologous MSCs, is not HLA-restricted, and is mediated by both cell–cell contact and soluble factors [43,44]. The reduction in T cell proliferation has been attributed, at least in part, to apoptosis [45]; the latter being associated with the expression of Fas ligand (FasL) in MSCs [46], although this view is not unanimous [47] (Figure 1).

Via interaction with dendritic cells, MSCs can induce the shift of CD4^+^ T cells with Th1 phenotype to cells with Th2 phenotype (Figure 1) and this is accompanied by downregulation of proinflammatory cytokines such as IL-6, IFN-γ, and IL-17 and by concomitant upregulation of the anti-inflammatory cytokines IL-4 and IL-10 [48,49]. Indoleamine 2,3-dioxygenase (IDO), which is constitutively produced by MSCs and is also induced following ΙFN-γ stimulation, seems to mediate, at least in part, these effects [49,50,51] (Figure 1). Additionally, via prostaglandin E2 (PGE2) secretion, MSCs suppress the differentiation of CD4^+^T cells into Th17 cells and inhibit their function in vitro [52] (Figure 1). MSCs may also impair IL-17 secretion by Th17 cells in a cell–cell-dependent mechanism and induce their conversion into T-regulatory cells (Tregs) [51,53] (Figure 1). In regard to Tregs, several studies have shown that MSCs promote their expansion and suppressive function and induce their generation from conventional T cells [51,54,55]. Implicated molecules in these processes include PGE2, transforming growth factor-β (TGF-β), hepatic growth factor (HGF) IL-10, and HLA-G [51,56,57] (Figure 1).

As far as B cells are concerned, MSCs inhibit their proliferation and activation via division arrest anergy [2,58,59] (Figure 1). Furthermore, MSCs have been shown to impair B cell maturation and antibody secretion, modulate the chemotactic properties of B cells [2,51,59,60], and induce the formation of B-regulatory cells (Bregs) [59] (Figure 1). These effects have been shown to involve cell–cell contact and soluble factors including IDO, PGE2, TGF-β, IL-1 receptor antagonist (IL-1RA), and IL-35 [51,61]. On the other hand, and despite the numerous studies reporting the suppressive effects of MSCs on B cells, others argue that MSCs can actually support the survival, proliferation, and differentiation of B cells to antibody-secreting cells [2,51,59,62] (Figure 1). This discrepancy can be explained by taking into consideration the fact that the effects of MSCs on B cells seem to be dependent upon the strength of their activation resulting from the inflammatory cues in the environment. To this end, when MSCs are inadequately inflammation-activated, they exhibit stimulatory effects but not inhibitory effects on B cells [39,51,63] (Figure 1).

MSCs have also been reported to induce the polarization of monocytes/macrophages towards an anti-inflammatory (M2) phenotype [39,51,64]. This seems to be associated with various soluble factors including IDO, HGF, IL-1RA, PGE2, TGF-β, and tumor necrosis factor-inducible gene 6 protein (TSG6) [51] (Figure 1). Anti-inflammatory monocytes secrete high levels of IL-10 but decreased levels of TNF-α, IL-12, IL-1β, and IL-17 [39,51,64,65] (Figure 1). A recent study showed that MSCs can also suppress monocyte functions [66] (Figure 1). More precisely, when human MSCs derived from the umbilical cord were cultured with human monocytes, the latter had decreased potential to differentiation into macrophages, defective phagocytic capacity, and antigen-presenting potential.

The immunoregulatory effects of MSCs also include dendritic cells (DCs) (Figure 1). More specifically, MSCs have been reported to suppress differentiation of human blood monocytes and cord blood CD34^+^ hematopoietic progenitor cells into DCs [2,41,43,51]. Furthermore, MSCs reduce the expression of HLA-DR, CD40, OX40L, CD80, CD83, and CD86 by DCs thereby decreasing their ability to stimulate T-cell proliferation [67,68]. MSCs decrease the production of IFN-γ IL-12 and TNF by DCs, whereas they induce IL-10 production, thereby impairing their ability for antigen presentation [2,42,51,63,69]. Collectively, these findings suggest that MSCs direct DCs to acquire a tolerogenic phenotype [2]. Several mechanisms have been implicated herein including interactions between Jagged1 on MSCs and Notch2 on DCs [51,70] as well as soluble factors such as PGE2, IL-6, TSG6, macrophage-colony-stimulating factor (M-CSF), and HGF [42,51,67,71].

With reference to natural killer (NK) cells (Figure 1), MSCs have been reported to downregulate their proliferation, cytotoxicity, and cytokine secretion via decreased production of IL-2 and IL-15 [2,72,73,74]. In addition, MSCs have been shown to decrease the expression of the surface receptors NKp44, NKp30, and NKG2D which are implicated in NK cell activation and cytotoxicity [74]. These effects are associated with soluble factors such as PGE2, TGF-β, IDO and soluble HLA-G and nitric oxide (NO) [2,63,72,73,74]. On the other hand, it has also been demonstrated that MSCs may be lysed by both autologous and allogeneic IL-2-stimulated NK cells [2,73] (Figure 1). This has been attributed to the fact that MSCs exhibit reduced expression of HLA-I molecules as well ligands which are recognized by activating NK receptors eventually triggering NK-cell mediated cytotoxicity [2,73,74]. Of note, MSC incubation with IFN-γ increased HLA-I expression and thus protected them from NK cell lysis [73].

## 3. MSCs’ Mode of Action

Because of their multipotency and immunoregulatory features, MSCs have attracted much attention in the field of regenerative medicine. It was initially thought that following adoptive transfer, MSCs would hone to the injured or damaged sites, engraft and subsequently restore defects by giving rise to mature functional cells [32]. However, although numerous studies have shown selective homing of transplanted MSCs to either injured or inflamed tissues [75,76], the levels of engraftment and differentiation are in most cases rather low to contribute physically to tissue regeneration to a significant extent [2,77,78,79]. Instead, many preclinical studies [80,81,82,83,84] suggested that the observed therapeutic impact of MSCs’ adoptive transfer is not probably related to their engraftment but to a transient presence of these cells into damaged tissues. Hence, it is now widely accepted that MSCs favor tissue repair via the secretion of various soluble factors and the shedding of extracellular vesicles [79]. Τhe former include bioactive molecules, i.e., cytokines, chemokines, and growth factors with proliferative, anti-inflammatory/immunoregulatory, angiogenic, and anti-apoptotic properties [85]. The nature of the secreted molecules is determined by the origin of MSCs, the donor age, and more importantly the surrounding microenvironment. Table 1 summarizes the key soluble factors produced by MSCs and the biological processes they are implicated in.

Aside from the aforementioned bioactive molecules, extracellular vesicles are increasingly being recognized as crucial mediators of MSCs’ biological functions. This concept has been supported by the fact that ΜSC-derived EVs can mimic the biological properties of their parental cells [109] and can exert similar anti-inflammatory, antiapoptotic, proangiogenic, and immunomodulatory effects in various disease models [110,111]. In the next section, the biology of EVs will be discussed and the immunomodulatory role of MSC-EVs will be explored.

## 4. Characterization of Extracellular Vesicles (EVs) and Immunomodulatory Properties of MSC-Derived EVs

Extracellular vesicles (EVs) are small membrane vesicles (30 nm to 4 μm in diameter), that are secreted by practically all eukaryotic cells [112,113]. Based on their size, composition, and biogenesis EVs are traditionally divided into three major subtypes: exosomes (50–150 nm diameter), microvesicles (100–1000 nm diameter), and apoptotic bodies (50–4000 nm diameter) [114,115]. In general, exosomes are formed within multivesicular bodies (MVB) and secreted following the fusion of the latter with the plasma membrane [115]. In contrast, microvesicles are directly formed and released from the plasma membrane via budding [5,115]. Similarly, apoptotic bodies are directly formed and released by cell membrane following cellular apoptosis [5,115].

Although EVs were initially considered as cellular debris devoid of any biological function, rapidly accumulating data has provided evidence that they are actually crucial effectors of intercellular communication, in both physiological and pathological conditions [116], via the transfer of their cargo. The latter consists of various proteins, lipids, and nucleic acids, the delivery of which can modulate the properties and functions of target cells [116,117].

Within this context, MSC-derived EVs have emerged as key mediators of the cells’ paracrine effects. In support of this notion, ΜSC-derived EVs have been shown to retain the biological functions of the cells they originate [109] and exert similar biological activity to the latter, including immunoregulation [110,111]. As far as the immunomodulatory role of MSCs-EVs is concerned, there is now robust evidence to suggest that they play a major part in exerting the effects of MSCs on the components of both innate and adaptive immunity. This issue will be briefly reviewed herein. For a more detailed discussion, the interested reader is referred to a recent comprehensive review by Bazzoni et al. [69].

In regards to the innate immune system, MSC-EVs have been demonstrated to inhibit DC activation eventually resulting in reduced triggering of T-cell responses ([69], and references therein). For example, Favaro et al. [118] showed that DCs from patients with type 1 diabetes treated with heterologous MSC-EVs acquired an immature phenotype, associated with decreased expression of activation markers and higher production of IL-6, IL-10, TGFβ, and PGE2. MSC-EV inhibition of DC maturation has been associated with upregulation of the microRNA (miRNA) miR-146 [119] and HLA-G [69,120]. Furthermore, it has recently been shown that MSC-EVs may also downregulate antigen uptake by immature DCs [69,121,122]. In terms of their role on NK cells, MSC-EVs have been found to inhibit their proliferation and IL-2 induced activation as well as their degranulation [69,123]. These effects could be mediated via MSC-EV expression of TGF-β, IL-10, and HLA-G [69,124].

Concerning MSC-EV effects on monocytes, recent data suggests that they inhibit the activation of the pro-inflammatory M1 macrophages, while concomitantly promote the activation of the anti-inflammatory M2 macrophages, thereby modifying the M1/M2 balance [6,69,125]. M2 macrophage polarization has been attributed to MSC-EV-induced up-regulation of S1P/SK1/S1PR1 signaling [69,126], innate immune signal transduction adaptor (MYD88), toll TLR signaling (TLR) [69,127], and, in the setting of lipopolysaccharide (LPS)-primed MSCs, LPS-dependent nuclear factor kappa B (NF-κB) signaling [69,128]. Furthermore, the potential of MSC-EVs to modulate the expression of chemokines (i.e., C-X-C motif chemokine ligand 1(CXCL1), C-C motif chemokine ligand (CCL5), CXCL2 ligand)) has been suggested to be involved in the down-regulation of M1 macrophage activation and inflammatory response ([69], and references therein). Furthermore, via the C-C motif chemokine receptor-2 (CCR2) expression, MSC-EVs bind and reduce the concentration of free CCL2 (CCR2 ligand) and thus inhibit the capacity of the latter to activate or recruit macrophages [69,129]. Various miRNAs have also been implicated in MSC-EV mediated M1/M2 imbalance ([69] and references therein), including miR-223, miR-155, miR-21, miR-146a in the setting of IL-1b primed MSCs, miR150-5p in the setting of IFN-γ-primed MSCs, and miR-let7. For instance, miR-let7 has been demonstrated to promote M2 macrophage polarization via the miR-let7/HMGA2/NF-κB pathway as well as macrophage infiltration via miR-let7/IGF2BP1/PTEN signaling [69,130]. In addition, LPS primed-MSC-EVs can modulate M1/M2 balance more efficiently than untreated MSCs and this has been attributed to the upregulation of let-7b expression which downregulates TLR4/NF-κB/STAT3/AKT regulatory signaling pathway, eventually restraining inflammation and promoting diabetic cutaneous wound healing [69,131]. Moreover, MSC-EVs inhibit M1-macrophage infiltration in injured/inflamed tissues by diminishing monocyte chemoattractant protein-1 (MCP-1), CCL5, high mobility group box protein 1 (HMGB1), and macrophage inflammatory protein 1α (MIP-1α), likely via miR-147 expression [69,132].

MSC-EVs have also been demonstrated to inhibit the proliferation of B cells [133] and to down-regulate B cell viability, the latter being associated with miR-155-5p [69,134]. The down-regulation of B cell proliferation is prominent following MSC-EV inflammatory priming [69,134]. In addition, MSC-EVs can inhibit B cell immunoglobulin production [69,135] and can also diminish the maturation of CD19^+^CD27^+^ memory B cells [69,125].

In an experimental immune encephalitis mouse model [69,136], MSC-EVs were demonstrated to produce programmed death-ligand 1 (PD-L1), galectin-1, and TGF-β1. Moreover, they were shown to inhibit auto-reactive T lymphocyte proliferation and induce T-cells to produce TGF-β1 and IL-10 [69,136]. MSC-EVs have also been found to up-regulate the generation of Tregs and this has been associated with increased IL-10 levels [137] and the upregulation of miR155, miR-let-7b, and miR-let7d ([69] and references therein) and to induce activated T-cell apoptosis [69,136]. Furthermore, MSC-EVs have been reported to suppress both CD4^+^ and CD8^+^ cells and to inhibit differentiation into effector and memory cells [69,138]. On the other hand, EVs released following cytochalasin B treatment from human adipose tissue-derived MSCs, genetically modified to overexpress interleukin-2 (IL2), were shown to activate and stimulate the proliferation of T-killer cells, which in turn were able to induce apoptosis in breast cancer cells [139]. In regards to other T-cell subsets, MSC-EVs have been reported to prevent Th17 cell development and IL-17 production [69,137] and to induce Th1 to Th2 shift [69,140].

## 5. The Impact of MSCs’ and MSC-EVs’ as Novel Therapeutic Modalities for Autoimmune Diseases

Autoimmune disorders represent a major cause of poor quality of life, morbidity, and increased healthcare costs [141]. Existing conventional therapies often require long-term administration and can be associated with significant toxicity and side-effects [141], while they may prove ineffective in a non-negligible proportion of patients [142]. Thus, it is imperative to develop alternative and more effective therapeutic modalities. Within this context, because of their multiple immunoregulatory effects, MSCs have emerged as an attractive novel therapeutic strategy for the treatment of autoimmune disorders.

Nevertheless, there are still some issues that need to be considered before the widespread application of this cell-based treatment. More precisely, even though human BMSCs have been shown to expand in vitro with no signs of immortalization [143,144], it must be borne in mind that the extensive ex vivo expansion, which is often necessary to produce adequate cell numbers for therapeutic purposes, carries the theoretical risk for clonal selection and subsequent malignant transformation [2,145]. Another potential risk associated with MSC-based therapy is their tumor-promoting potential [2] (reviewed in [146]), which has been demonstrated in several animal tumor models (reviewed in [146]). However, to the best of our knowledge, no tumor formation has been reported thus far in human subjects who received MSCs [147], although a more extended follow-up may be required, to accurately assess their potential tumorigenic capacity.

To this end, MSC-derived EVs may represent a more advantageous approach as compared to MSC-based treatment [5]. While they retain the properties and functions of their parental cells [109], MSC-EVs do not seem to be associated with the aforementioned risks and concerns and are considered safer than MSC treatment [5,8]. More precisely, MSC-EVs are unable to proliferate and have not been shown to promote tumor growth [5,109,148]. Furthermore, MSV-EVs cannot differentiate and thus can bypass the risk of ectopic bone formation at the site of injection previously reported for MSCs [149,150]. Moreover, they are associated with minimal immunogenicity [109,151] and possess the advantage of being able to cross the blood–brain barrier [152]. These advantages have set the stage for investigating the therapeutic potential of MSC-EVs in various diseases including immune-mediated disorders [8].

**Figure 2:**
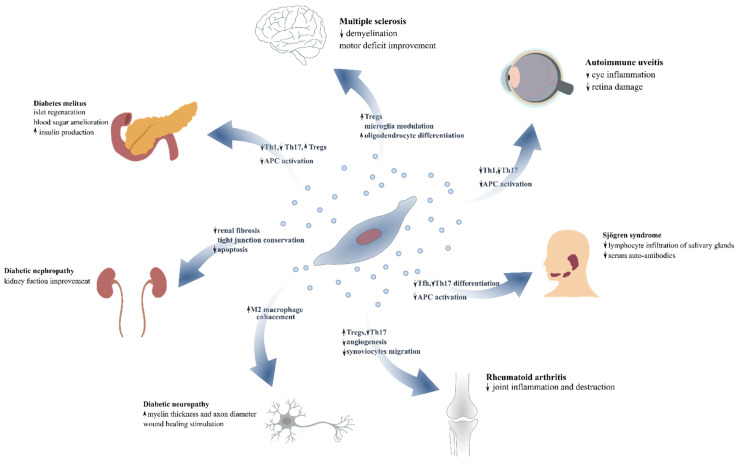
Overview of the impact of MSC-derived EVs in autoimmune disorders. MSC: mesenchymal stromal cells; EV: extracellular vesicles.

In the next sections, we will summarize findings from in vitro studies, experimental animal models, and pioneering clinical trials of MSCs and their EVs (Figure 2) in the setting of autoimmune diseases.

## 6. Diabetes Mellitus

Diabetes mellitus (DM) is a leading cause of morbidity and mortality worldwide with glycemic dysregulation resulting in a variety of complications in diabetic patients [153]. The two clinically distinct types of DM are characterized by different pathophysiologic mechanisms. While type 2 diabetes is associated with insufficient production and insulin resistance as well as chronic low-grade inflammation, type 1 diabetes is merely caused by autoimmune destruction of insulin-producing pancreatic islet β-cells [154,155].

Insulin replacement represents the standard treatment modality for type 1 DM patients. However, it is not sufficient to prevent long-term disease complications [155] and thus the need for the development of alternative therapies seems mandatory. Within this context, and in view of the autoimmune nature of type 1 DM, MSCs have emerged as an appealing novel approach thanks to their multiple immunomodulatory and regenerative effects.

Indeed, MSC-based therapy has shown encouraging results in both preclinical and clinical studies. In regard to the latter, Moreira et al. [156] recently reviewed existing clinical data on the therapeutic use of MSCs in both type 1 and type 2 DM and concluded that this approach is well-tolerated and potentially beneficial. Various research groups have actually observed improvement of glycated hemoglobin (HbA1c), C peptide levels, and insulin dosage requirement upon treatment with autologous and allogeneic MSCs, suggesting a possible restoration of islet β-cells in patients with DM [157,158,159,160].

As far as MSC-EVs are concerned, various studies (Table 2) have recently provided evidence for a potential therapeutic role in DM by modulating the immune microenvironment and sustaining β-cell regeneration [161]. In a mouse model of type 1 DΜ, MSC-EVs were shown to inhibit T cell proliferation and suppress antigen-presenting cell activation leading to the delay of disease onset [162]. In another report, co-culturing CD14^+^ cells derived from patients with type 1 DM with MSC-EVs in vitro switched their differentiation to IL-10-secreting DCs [118]. Subsequently, these conditioned DCs led to an inhibition of Th17 cells and an increase in Tregs [118]. These findings merit further investigation in view of a study in type 1 DM mice [163], in which intraperitoneal administration of autologous adipose tissue-derived MSC-EVs shifted the cytokine profile towards anti-inflammation and induced upregulation of Tregs, thereby resulting in alleviation of disease-related pathological, immunological, and clinical parameters. With respect to MSC-EVs’ β-cell regenerative action, it has recently been demonstrated that intravenous injection of EVs derived from menstrual blood-MSCs increased β-cell mass and insulin production in a type 1 DM rat model [164]. Immunohistochemistry analysis corroborated the presence of insulin in the islets of treated animals [164]. It was suggested that MSC-EVs’ regenerative effects were mediated via the pancreatic and duodenal homeobox 1 pathway. Wen et al. [165] investigated the role of exosomes in improving the outcome of islet transplantation. As the destruction of transplanted islets can be attributed to immune rejection in which miR-375 and FAS are implicated, the authors transfected human BM MSC-derived exosomes with a plasmid encoding for shFas and anti-miR-375. Administration of these exosomes induced downregulation of Fas and miR-375 in human islets and improved the survival and function of islet allograft in diabetic mice [165].

## 7. Diabetic Complications

Wound-healing impairment and diabetic ulcers are major complications in diabetic patients that cause significant impairment in the quality of life [182,183]. In a mouse model of diabetic foot ulcers, stimulation of wound healing was achieved by MSC-EV treatment [166] (Table 2). More specifically, MSC-EVs overexpressing the long non-coding RNA H19 (lncRNA H19), which is known to exert beneficial effects on the regulation of endogenous glucose production in diabetic hyperglycemia, were injected into the surrounding tissues of the wound. This resulted in the induction of proliferation and migration of fibroblasts with concomitant inhibition of apoptosis. Mechanistically, these effects were associated with the down-regulation of miR-153-by lncRNA H19, which in turn resulted in the upregulation of the phosphatase and tensin homolog (PTEN) phosphatase [166].

In a rabbit model of diabetic retinopathy intravenous, intraocular, or subconjunctival administration of adipose tissue-derived MSC-EVs led to retina protection [167] (Table 2). This was associated with the transfer of miR-222, and negative regulation of angiogenesis from EVs to retinal cells. Additionally, intravitreal administration of umbilical cord-derived MSC-EVs expressing miR-126, previously shown to improve diabetic retinopathy [60], reduced inflammatory cytokine production in the vitreous humor in diabetic rats via inhibition of HMGB1 [161,168] (Table 2).

The beneficial effects of MSC-EVs have also been demonstrated in another diabetic complication, namely neuronal degeneration (Table 2). In this context, administration of BM-MSC-EVs resulted in a switch of the immune equilibrium towards M2 macrophages which was associated with increased nerve thickness and concomitant functional recovery in a diabetic peripheral neuropathy mouse model [169]. In another study [170] (Table 2), intravenous or intracerebroventricular injection of BM-MSC-EVs improved diabetes-induced cognitive impairment via inhibition of oxidative stress and an increase in synaptic density in a murine model of type 1 DM.

Regarding diabetic nephropathy (Table 2), ΒΜ-MSC-EVs have been shown to decrease renal fibrosis in diabetic mice [120], conserve tight junction between tubular epithelial cells, and exert antiapoptotic and anti-inflammatory effects thus improving clinical parameters associated with renal function [184]. Autophagy induction by MSC-EVs was also postulated as a possible mechanism of renal protection [185]. In a human pioneering clinical trial [186], 20 patients with chronic kidney disease, 10 of whom were diabetic, received intra-arterial MSC-EVs. The treatment resulted in kidney function improvement with an increase in estimated glomerular filtration rate (eGFR).

## 8. Rheumatoid Arthritis

Rheumatoid arthritis (RA) is a common autoimmune disease with a complex pathophysiologic background involving genetic, epigenetic, and environmental components. The imbalance of innate and adaptive immunity pathways has been implicated in causing synovial inflammation, thereby leading to characteristic features of chronic polyarthritis [187].

At present, therapies for RA include non-steroidal anti-inflammatory drugs, corticosteroids, anti-rheumatic drugs, and biological factors. Despite the effectiveness of these approaches in the majority of patients, a significant number of them experiences adverse effects while some RA patients are resistant to these therapies.

MSCs-based treatment has been considered as a promising therapeutic approach in RA [188]. Administration of human umbilical cord-derived MSCs, BM-MSCs, and MSCs derived from exfoliated deciduous teeth led to a reduction in bone erosion, examined by micro-CT imaging, and alleviation of synovitis and articular destruction with concurrent clinical amelioration in a RA mouse model [189]. Notably, umbilical cord MSCs have been shown to inhibit joint inflammation and bone erosion, while supporting cartilage formation in mice, and these immunomodulatory effects have been associated with the inhibition of inflammatory cytokines (IL-1, IL-6) and the expansion of Tregs [190]. In line with these findings, the administration of adipose tissue-derived MSCs suppressed Th17 differentiation and prompted the generation of IL-10-secreting Tregs resulting in clinical amelioration of RA mice [191]. Polarization of naive macrophages toward an M2 phenotype [192] and inhibiting the activation of DCs and NK cells by MSCs has additionally been suggested in RA animal models [193].

In line with the experimental RA models, clinical studies have reported encouraging results concerning the safety and efficacy of MSC-based treatment in patients with RA. To this end, a study conducted by Álvaro-Gracia et aI. [194] showed that the infusion of allogeneic adipose tissue-derived stem cells in a cohort of patients with refractory RA was generally well-tolerated with most adverse events ranging from mild to moderate. Likewise, in a smaller phase I trial evaluating the use of umbilical cord-derived MSCs, Park et al. [195] reported no major adverse events. Moreover, the authors observed an improvement in disease activity scores and a decrease in pro-inflammatory cytokines 24 h post infusion [195]. In a triple-blind, placebo-controlled phase 1/2 clinical trial, Shadmanfar et al. [196] randomized RA patients with knee involvement to receive either intra-articular injection of autologous bone marrow-derived MSCs or normal saline (placebo). MSC administration had no adverse events and was associated with better clinical outcomes, which, however, could not be maintained beyond 12 months, with the exception of improved standing time [196]. Additionally, a reduction in methotrexate and prednisolone intake was observed in the MSC group for the first 6 months of follow-up [196]. In another study, Ghoryani et al. [171] provided evidence for the amelioration of clinical findings in RA patients treated with autologous BM-MSCs, and this was associated with Th17 downregulation and Treg upregulation.

Due to their immunoregulatory and anti-inflammatory properties, MSC-EVs have also been explored as a novel therapeutic option in RA treatment in both in vitro and preclinical studies (Table 2). More specifically, incubation of umbilical cord-derived MSC-EVs with peripheral blood mononuclear cells from RA patients led to a decrease in the proportion of Th17 cells and in the production of IL-17 along with a concomitant upregulation in the proportion of Treg cells and TGF-β expression [197]. These immunomodulatory effects of umbilical cord-derived MSC-EVs provided the theoretical background for their potential application in RA treatment. Another study demonstrated that mouse BM-derived MSC-EVs increased Tregs and decreased CD4^+^ and CD8^+^ cells as well as plasmablast differentiation [172]. In RA mice, these BM-derived MSC-EVs were further shown to reduce clinical features of inflammation and this effect could be attributed to fewer plasmablasts and more Breg-like cells in the animals’ lymph nodes [172]. Chen et al. [173] investigated the effect of exosomes derived from BM-MSC previously transfected with miR-150-5p. This miRNA is implicated in T cell maturation and angiogenesis and is expressed at lower levels in RA patients as compared to controls [198,199,200]. The aforementioned exosomes inhibited migration and invasion of fibroblast-like synoviocytes (FLS) from RA patients. Moreover, they reduced angiogenesis, joint inflammation, and clinical arthritic scores in vivo in a murine RA model [173]. These effects were associated with the downregulation of matrix metalloproteinase-14 (MMP14) and vascular endothelial growth factor (VEGF) expression. These molecules, which are increased in RA, are involved in disease development, joint damage, and synovial inflammation [172]. Following a similar experimental approach, Zheng et al. [174] transfected EVs derived from rat BM-MSCs with miR 192-5p which suppresses the growth of RA-FLSs. Injection of the MSC-EVs in RA rats resulted in a reduction in synovial hyperplasia and joint destruction via suppression of pro-inflammatory cytokines. This was attributed to the downregulation of Ras-related C3 botulinum toxin substrate 2 (RAC2) by the EV miR 192-5p [174].

## 9. Sjogren’s Syndrome-Autoimmune Sialadenitis

Sjogren’s syndrome (SjS) is a systemic autoimmune disease, which mainly affects salivary and lacrimal glands and results in mucosal dryness [201,202]. B and T cell infiltration, as well as autoantibody production against Ro/SSA and La/SSB antigens, are the main features of the immune dysregulation that mediate epithelial destruction of the exocrine glands [201,202].

The role of MSCs in SjS has been investigated in both animal and human studies. Hence, in an SjS-like mouse model, Xu et al. [202] showed that administration of mouse BM-MSCs improved salivary gland secretory function by inducing CD4^+^ T cells to differentiate towards Treg and Th2 cells and by downregulating Th17 and T follicular helper (Tfh) inflammatory responses. Based on these results, the authors further evaluated the safety and efficacy of allogenic umbilical cord-MSC treatment in 24 SjS patients. Interestingly, all patients showed clinical improvements as well as a significant reduction in serum levels of anti-Ro/SSA and anti-La/SSB, while no adverse events were observed during or after the infusion of umbilical cord-MSCs [202].

Another study [175] comparatively addressed the role of induced pluripotent stem cells (iPSC)-derived MSCs and their EVs, as well as BM-MSCs in a sialadenitis preclinical murine model (Table 2). iPSC-MSCs, administered before or at the very beginning of sialadenitis, did not differ from BM-MSCs in their capacity to reduce lymphocyte infiltration into mouse salivary glands and eventually delayed SjS progression. Furthermore, iPSC-MSC-derived EVs suppressed inflammatory cells and inhibited the expression of proinflammatory factors in vitro with the same efficacy as EVs derived from BM-MSCs. In addition, infusion of EVs derived from iPSC-MSCs led to decreased lymphocyte infiltration in salivary glands and reduced serum autoantibodies, albeit to a lesser extent as compared to iPSC-MSCs and BM-MSCs [175]. These results support the emerging role of EVs in preventing SjS before the onset of sialadenitis.

## 10. Autoimmune Uveitis

Autoimmune uveitis is an inflammatory disease involving the vascular layer of the eye leading to visual impairment and even blindness. Autoimmune uveal inflammation may occur as an isolated entity or it may be associated with systemic autoimmune syndromes [203]. Current treatments include the use of corticosteroids and other immunosuppressants as well as biologic agents. However, these therapeutic modalities may be associated with significant local and systemic side effects when applied for a prolonged period of time [203]

MSC-based treatment has shown promising results in experimental autoimmune uveitis (EAU), a T cell-mediated autoimmune disease characterized by ocular inflammation, destruction of the retinal architecture, and photoreceptor cell layer that represents a well-established animal model of human uveitis [204,205,206,207]. More specifically, Zhang et al. [205] demonstrated that intravenous administration of BM-MSCs in EAU rats resulted in delayed disease onset and reduced disease severity. These effects were associated with a decreased Th17/Treg ratio and reduced proinflammatory cytokine production (IL-2, IFN-γ, IL-6, and IL-17), with a concomitant increase in anti-inflammatory cytokine (IL-10, TGF-β) levels [205,207]. In another study, Ko et al. [208] reported the mitigation of inflammatory infiltration and clinical findings in mice with EAU after intravenous injection of human MSCs. These effects are mediated by an increased monocyte/macrophage population (MHCII^+^, B220^+^, CD11b^+^) in peripheral blood, spleen, and draining lymph nodes exhibiting suppressive effects on T cell proliferation and Th1/Th17 differentiation [208]. Similar results have been obtained with intraperitoneal injection of MSCs in EAU mice. Indeed, Tasso et al. [206] observed disease improvement, which was associated with systemic Treg expansion in EAU mice receiving syngeneic MSCs. Furthermore, Oh et al. [209] reported that intraperitoneal administration of human MSCs suppressed Th1 and Th17 cells and increased B220^+^CD19^+^ cells expressing IL-10 in draining lymph nodes with a concomitant decrease in proinflammatory cytokines in the eyes in EAU mice.

MSC-EVs have also shown beneficial results in the treatment of EAU treatment (Table 2). To this end, periocular injection of EVs derived from human umbilical cord MSCs reduced leukocyte infiltration, protected retinal structure, and rescued retinal function in a rat model of autoimmune uveitis [176]. The attenuation of ocular inflammation by EVs was associated with the downregulation of CD4^+^INFγ^+^ and CD4^+^IL17^+^ cells in the retina. Furthermore, inhibition of chemotactic effects of CCL2 and CCL21 on inflammatory cells was demonstrated in an in vitro assay and was proposed as an additional mechanism mediating the immunosuppressive action of MSC-EVs [176]. Likewise, intravenous injection of human MSC-EVs reduced CD3^+^ cell infiltration, suppressed proinflammatory cytokines in the eyes, and decreased IFN-γ^+^CD4^+^ and IL-17^+^CD4^+^ cells in cervical draining lymph nodes in a mouse model of EAU [162]. Moreover, in an in vitro mixed lymphocyte reaction, MSC-EVs exhibited inhibitory effects on Th1 and Th17 cells and also suppressed co-stimulatory factors and HLA-II expression of antigen-presenting cells [162].

## 11. Multiple Sclerosis—Experimental Autoimmune Encephalitis

A large body of evidence has accumulated during the last years regarding the use of MSCs and their EVs in the treatment of neurodegenerative diseases with autoimmune features such as multiple sclerosis (MS) [210]. MS is a chronic inflammatory demyelinating disease of the central nervous system (CNS) with a wide range of symptoms that represents one of the main causes of neurological deficits in young adults [211,212].

Various studies have been reported on the beneficial role of MSCs in MS experimental models such as Experimental Autoimmune Encephalomyelitis (EAE) [213]. Thus, MSCs have been shown to alleviate EAE manifestations primarily by regulation of T cell-mediated immune mechanisms [214], suppression of Th1 and Th17 cells, and induction of macrophage polarization from M1 to M2 phenotype, eventually resulting in a decrease in lymphocyte infiltration and nerve demyelination [213]. With regards to clinical trials, studies published thus far have reported the use of autologous BM-MSCs and conditioned media, adipose tissue-MSCs, umbilical cord-MSCs, and neural progenitors derived from autologous BM-MSCs in MS patients (reviewed in [213]). Because of the small number of included patients, many of these trials mainly provided evidence only for the feasibility and safety of MSC treatment, globally reporting favorable results (reviewed in [213]). There have also been a few studies suggesting that administration of MSCs may have beneficial effects in MS patients [215,216,217], however, these data require further confirmation (reviewed in [213]).

EVs from human bone marrow-MSC have also been shown to ameliorate clinical outcomes in EAE murine studies (Table 2). Hence, a significant reduction in demyelination and inflammation was observed in mice treated with MSC-EVs [177]. These findings were associated with an increase in CD4^+^CD25^+^FOXP3^+^ Tregs in the spinal cord and the suppression of pro-inflammatory cytokines. These effects were even more pronounced in IFN-γ-primed MSC exosomes, suggesting their potential role in the treatment of autoimmune neurodegenerative diseases [177]. Another study [178] demonstrated that administration of EVs derived from human adipose tissue-MSCs in EAE mice reduced the clinical score and myelin oligodendrocyte glycoprotein-induced proliferation of splenocytes. In addition, demyelination areas and inflammatory infiltrates decreased significantly in EV-treated animals [178]. Using a Theiler’s murine encephalomyelitis virus (TMEV)-induced demyelinating disease as a model of progressive MS, Laso-García et al. [179] reported that administration of EVs derived from adipose tissue-MSCs improved motor status, brain atrophy, proliferation in the subventricular zone cells, and decreased inflammatory infiltrates in the mice spinal cord of mice. In addition, treatment with EVs was also able to decrease plasma cytokine levels, mainly in the Th1 and Th17 phenotypes. [179]. In another study [180], EVs derived from placental MSCs were shown to protect oligodendrocytes from damage and to increase myelination in the spinal cord. In vitro evidence suggested that the beneficial effects of EVs in myelination were associated with the induction of differentiation of endogenous oligodendrocyte precursors to mature myelinating cells [180]. Finally, a recent report [181] has shown that MSCs primed with INF-γ produce EVs that are able to dampen the pro-inflammatory phenotype of microglia cells. This could be mediated via EVs’ miRNA cargo. More specifically, miR-467f and miR-466q have been demonstrated to exert an immunomodulatory effect on microglia by inhibiting the expression of Map3k8 and Mk2 and thus downregulating the p38 MAPK signaling pathway [181]. Of note, intravenous or intraperitoneal administration in EAE mice of EVs derived from MSCs primed with INF-γ decreased proinflammatory markers in the spinal cord of the animals. Overall, these data suggest that MSC-derived exosomes may also affect neuroinflammation through specific immunomodulatory miRNAs acting on microglia [181].

Additionally, the immunomodulatory effect of MSC-EVs in the brain has been directed in different inflammatory scenarios, such as neonatal brain injury, with similar effects. As it has been recently reviewed by Matei et al. [218], MSC-EVs from different sources have been found to restore blood–brain barrier integrity, modulate microglia activation, reduce apoptosis, and reduce white matted loss in a variety of experimental settings of hypoxic-ischemic encephalopathy caused by perinatal oxygen deprivation [218].

## 12. Systemic Lupus Erythematosus

Systemic lupus erythematosus (SLE) remains probably one of the most complex autoimmune diseases with a wide spectrum of clinical manifestations and complex pathophysiology [219]. Current treatment of SLE includes antimalarials, glucocorticoids, nonsteroidal anti-inflammatory drugs, immunosuppressants, cyclophosphamide, and biologic agents [219]. However, a subset of patients is refractory to these agents, while their prolonged use may be associated with various side effects. During the past years, MSCs have emerged as potential candidates for the treatment of SLE because of their anti-inflammatory and immunomodulatory properties [220]. Their beneficial role in SLE has indeed been corroborated in both preclinical and clinical studies [220,221].

More specifically, in murine SLE models, human BM-MSC administration restored bone marrow microenvironment (osteoblastic niche) and reduced anti-nuclear and anti-double strand DNA antibody blood levels. Furthermore, MSC administration improved glomerular morphology/structure and diminished renal complex deposition of both complement component 3 (C3) and IgG [220,222]. These effects have been associated with the potential of BM-MSCs to induce B-cell suppression [223,224] and to inhibit Th17 and follicular T helper cell development with a concomitant restoration of Treg levels [37,225].

With regard to the therapeutic potential of MSCs in refractory SLE patients, clinical studies (reviewed in [188,220,226]) have collectively demonstrated an acceptable safety profile with disease activity improvement and beneficial—-yet variable—effects on clinical remission (reviewed in [188,220,226].

The use of MSC-EVs is also being suggested in SLE as an alternative to MSCs, with which they share comparable immunomodulatory effects [227]. In a mouse model of acute kidney injury, MSC-EVs reduced kidney inflammation and preserved kidney function [228] providing the theoretical background for their potential application in lupus nephritis. However, to our knowledge, no data has been published thus far addressing the role of MSC-EVs in SLE animal models and patients.

## 13. Conclusions

During the last decades, the immunomodulatory properties of MSCs have been extensively investigated and a rapidly growing number of studies has provided substantial evidence for the safety, tolerability, and efficacy of MSCs in an autoimmune disorders setting. Although MSC-based therapies hold great promise for the treatment of immune-mediated diseases, variability regarding the origin of MSCs, the age and sex of the donor, isolation and expansion protocols, cell dose, mode, and schedule of administration have resulted in inconsistent results, thereby hindering translation into daily practice. Furthermore, despite the well-established safety profile of MSC treatment, there have been several concerns affecting its widespread clinical application such as the theoretical risk of tumorigenicity, genomic instability, and unwanted differentiation [2].

On the other hand, it is now widely acknowledged that MSC-EVs are key mediators of the immunoregulatory effects of MSCs affecting both innate as well as adaptive immune responses. While they exert similar immunomodulatory functions as their parental cells, MSC-EVs cannot proliferate nor differentiate and do not, therefore, raise many of the concerns associated with stem cell therapy. Hence, MSC-EVs have drawn much attention over the last years as an alternative, cell-free therapy, for the treatment of autoimmune disorders. Accumulating data, mostly preclinical, has supported this notion and has provided the rationale for exploring the therapeutic efficacy of MSC-EVs more deeply. To this end, unraveling the underlying molecular and cellular mechanisms mediating the beneficial effects of MSC-EVs is eagerly anticipated along with the design of clinical trials assessing long-term safety and outcomes.

However, to properly assess the effects of MSC-EVs in the clinical setting, while minimizing controversies, general consensus should be reached regarding the optimal protocols for isolation, purification and characterization, quantification, and storage of MSC-EVs. Furthermore, the yield of generated MSC-EVs needs to be increased for their use in clinical trials, and this is currently being pursued by testing various modifications of culture conditions and manipulations of MSCs to increase EV production [8]. One such promising approach includes MSC treatment with Cytochalasin B, which results in the production of membrane vesicles (CIMVs) [229] that contain the cytoplasmic content of MSCs and retain their immunophenotype, biological activity, and immunosuppressive properties of the latter. Finally, the optimal dosage and therapeutic schedule of MSC-EVSs’ administration should be determined as well as assays to accurately assess their efficacy [5,8,230].

Addressing these issues will greatly contribute to our understanding of the potential of MSC-EVs in immune-mediated diseases and other disorders and provide a robust theoretical background for translating this therapeutic modality into the clinical setting.

## Figures and Tables

**Table 1:** Soluble factors secreted by MSCs and their biological functions.

Soluble Factors	Biological Functions	References
ITGα1, ITGα3, ITGα4 ITGα5, CD44, Galectin, CXCL12	Cell attachment, spreading, proliferation, differentiation	[86,87,88,89,90]
MT1-MMP, MMP1, MMP9, TIMP1, TIMP2, TIMP4, PA, TNF-α	Cell–cell adhesion, tissue remodeling, cell migration, invasion, proliferation, apoptosis, differentiation, angiogenesis	[91,92,93]
CCR2, CCR7, CCR10, CXCR4, CXCR5, CXCR6	Migration, angiogenesis immunomodulation	[94,95]
HLA-G5, IDO, iNOS, IL-6, IL-10, LIF, PGE2, TGF-β	Immunomodulation	[35,42,51,96]
ANGs, FGF2, TGF-β, VEGF	Angiogenesis	[92,97,98,99,100,101,102,103]
FGF2, GM-CSF, IGF, SFRP1,SFRP-2,TGF-β	Cell survival and proliferation	[92,97,104,105,106,107,108]

ANGs: Angiopoietins, CCR: CC chemokine receptor, CXCL12: C-X-C motif chemokine ligand 12, CXCR: C-X-C motif chemokine receptor, FGF2: Fibroblast growth factor 2, GM-CSF: Granulocyte macrophage colony stimulating factor, HLA-G5: Human leukocyte antigen G5, IDO: Indoleamine 2,3-dioxygenase, IGF: Insulin-like growth factor, iNOS: inducible nitric oxide synthase, IL: Interleukin, ITG: Integrin, LIF: Leukemia inhibitory factor, MMP: Matrix metalloproteinase, MT-MMP: Membrane-yype matrix metalloproteinase, PA: Plasminogen activator, PGE2: Prostaglandin E2, SFRP: Secreted frizzled-related protein, TIMP: Tissue inhibitor of metalloproteinase, TGF-β: Transforming growth factor beta, TNF-α: Tumor necrosis factor-alpha, VEGF: Vascular endothelial growth factor.

**Table 2:** In vitro and preclinical studies of MSC-derived EVs in autoimmune diseases.

Disease	Experimental Model	EV Source	Administration Route	Effects	Outcome	Reference
**DM**	Mouse model, in vitro study	Human MSCs	iv injection	Suppression of Th1 and Th17, inhibition of APC activation	Delay of disease onset	[162]
**DM**	In vitro study	Human BM-MSCs		CD14^+^ cells’ differentiation to IL-10 secreating DCs, inhibition of Th17 and upregulation of Tregs		[118]
**DM**	Mouse model	Mouse AD-MSCs	ip injection	Increase in Tregs and anti-inflammatory cytokine	Pancreatic islet regeneration, blood glucose levels and body weight improvement	[163]
**DM**	Rat model	Human MenSCs	iv injection	Pancreatic and duodenal homeobox 1 pathway modulation	Increase in β-islet mass and insulin production	[164]
**DM**	Mouse model	Genetically manipulated human BM-MSCs	Human islets and MSCs co-culture	Downregulation of Fas and miR-375	Improvement of survival and function of islet allograft	[165]
**Diabetic** **u** **lcer**	Mouse model, in vitro study	Genetically manipulated mouse BM-MSCs	Intracutanous injection	Enhancement of fibroblast proliferation and migration	Stimulation of wound healing	[166]
**Diabetic retinopathy**	Rabbit model	Rabbit AD-MSCs	iv, subconjunctival and intraocular injection	Increase in miR-222 expression	Retina regeneration	[167]
**Diabetic** **r** **etinopathy**	Rat model	Manipulated human UC-MSCs	Intravitreal injection	Inhibition of HMGB1 signaling pathway	Alleviation of retinal inflammation	[168]
**Diabetic peripheral neuropathy**	Mouse model	Mouse BM-MSCs	iv injection	M2 macrophage phenotype enhancement	Increase in myelin thickness and nerve axonal diameter	[169]
**Cognitive impairment in diabetes**	Rat model	Rat BM-MSCs	iv, intracerebroventricular injection	Inhibition of oxidative stress and increases synaptic density	Cognitive improvement	[170]
**Diabetic nephropathy**	Mouse model	Human BM-MSCs	iv injection	Inhibition of renal fibrosis	Improvement of renal function	[120]
**RA**	In vitro study	human UC-MSCs		Increase in Treg/Th17 ratio, up-regulation of IL-17 and TGF-β		[171]
**RA**	Mouse model	Mouse BM-MSCs	iv injection	Increase in Tregs, decrease in CD4^+^ and CD8^+^ cells, suppression of Bregs and plasmablasts in lymph nodes	Decreased clinical signs of inflammation	[172]
**RA**	Mouse model	Genetically manipulated mouse BM-MSCs	ip injection 186	Inhibition of migration of fibroblast-like synoviocytes, down-regulation of angiogenesis	Amelioration of joint inflammation and clinical arthritis score	[173]
**RA**	Rat model	Genetically manipulated rat BM-MSCs	iv injection	Suppression of pro-inflammatory cytokines	Reduction in clinical arthritic scores, joint destruction, and inflammatory response	[174]
**Sjogren’s syndrome**	Mouse model, in vitro study	BM-MSCs, iPSC-MSCs	iv injection	Inhibition of Tfh and Th17 differentiation, APCs activation and expression of costimulatory mole-cules by salivary gland epithelial cells	Delay in lymphocyte infiltration into salivary glands and decrease in serum autoantibody levels	[175]
**Experimental autoimmune uveitis**	Rat model, in vitro study	UC-MSCs	Periocular injection	Inhibition of chemoattractive effects of CCL2 and CCL21 on inflammatory cells, reduction in infiltration of inflammatory cells in the eyes.	Reduced the intensity of experimental auto-immune uveitis	[176]
**Experimental autoimmune uveitis**	Mouse model, in vitro study	Human MSCs	iv injection	Inhibition Th1 and Th17, decrease in co-stimulatory factors and MHCII in APCs	Decrease in retinal structural damage	[162]
**Multiple sclerosis/experimental autoimmune** **e** **ncephalomyelitis**	mouse model	human BM-MSCs and UC-MSCs	iv injection	reduction of demyelination, decrease in neuroinflammation and upregulation Tregs within the spinal cord	amelioration of neurological clinical score	[177]
**Multiple sclerosis/experimental autoimmune encephalomyelitis**	Mouse model	Human AD-MSCs	iv injection	Reduction in T-cell proliferation, leukocyte infiltration and demyelination	Improvement in neurological clinical score	[178]
**Multiple sclerosis/Theiler’s murine encephalomyelitis virus-induced demyelinating disease**	Mouse model	Human AD-MSCs	iv injection	Modulation of activation state of microglia, reduction in proinflammatory cytokine levels in the plasma	Decreased inflammatory infiltrates in the spinal cord, reduction in brain atrophy and improvement in motor function	[179]
**Multiple sclerosis/experimental autoimmune encephalomyelitis**	Mouse model	Human P-MSCs	iv injection	Reduction in DNA damage in oligodendroglia, induction of oligodendrocyte precursors differentiation towards mature myelinating cells and increase myelination	improvement in motor deficit	[180]
**Multiple sclerosis/experimental autoimmune encephalomyelitis**	Mouse model, in vitro study	Mouse BM-MSCs	iv injection	Reduction in the pro-inflammatory phenotype of microglia cells attributed to miR-467f and miR-466q mediating downregulating of the p38 MAPK signaling pathway	Reduction in proinflammatory markers in the spinal cord of the animals but no effect on disease course	[181]

AD-MSC: Adipose tissue-derived MSCs, BM-MSCs: Bone marrow-derived MSCs, DCs: Dendritic cells, EAE: Experimental autoimmune encephalomyelitis, HMGB1: High mobility group Box 1, ip: intraperitoneal, iPSCs: Human-induced pluripotent stem cells, iv: intravenous, MenSCs: Menstrual blood-derived MSCs, miRNA: micro RNA, MSCs: Mesenchymal stromal cells, Th1: T helper 1 cells, Th17: T helper 17 cells, TfH: T follicular helper cells, APCs: antigen-presenting cells, Tregs: T regulatory cells, P-MSCs: placenta-derived MSCs, RAC2: Ras-related C3 botulinum toxin substrate 2, UC-MSCs: umbilical cord-derived MSCs.
